# Reconstruction of audio waveforms from spike trains of artificial cochlea models

**DOI:** 10.3389/fnins.2015.00347

**Published:** 2015-10-13

**Authors:** Anja T. Zai, Saurabh Bhargava, Nima Mesgarani, Shih-Chii Liu

**Affiliations:** ^1^Institute of Neuroinformatics, University of Zurich and ETH ZurichZurich, Switzerland; ^2^Department of Electrical Engineering, Columbia UniversityNew York, NY, USA

**Keywords:** spike reconstruction, cochlea spikes, silicon cochlea, cochlea model, digit recognition, analog reconstruction

## Abstract

Spiking cochlea models describe the analog processing and spike generation process within the biological cochlea. Reconstructing the audio input from the artificial cochlea spikes is therefore useful for understanding the fidelity of the information preserved in the spikes. The reconstruction process is challenging particularly for spikes from the mixed signal (analog/digital) integrated circuit (IC) cochleas because of multiple non-linearities in the model and the additional variance caused by random transistor mismatch. This work proposes an offline method for reconstructing the audio input from spike responses of both a particular spike-based hardware model called the AEREAR2 cochlea and an equivalent software cochlea model. This method was previously used to reconstruct the auditory stimulus based on the peri-stimulus histogram of spike responses recorded in the ferret auditory cortex. The reconstructed audio from the hardware cochlea is evaluated against an analogous software model using objective measures of speech quality and intelligibility; and further tested in a word recognition task. The reconstructed audio under low signal-to-noise (SNR) conditions (SNR < –5 dB) gives a better classification performance than the original SNR input in this word recognition task.

## 1. Introduction

Spiking neuromorphic sensors such as silicon retina and cochlea sensors produce outputs only in response to stimulus changes, in contrast to frame-based sensors where frames are generated periodically regardless of incoming signal. Their asynchronous outputs can carry more precise timing information about stimulus changes when compared to sampled outputs. Processing such asynchronous outputs can be useful for understanding the benefits of event-driven processing also seen in brains. For example, the pencil balancer system which uses the silicon Dynamic Vision Sensor (DVS) retina system (Lichtsteiner et al., 2008) showed lower response latency in the sensory-motor loop and 100X lower computational costs, both as a result of using the sparse and quick events from the DVS in response to the moving pencil (Conradt et al., 2009).

A further example is demonstrated for an auditory source localization algorithm that uses the output spikes of a silicon cochlea which are phase-locked to the input frequency. The sparser sampled events can lead to a reduction of 30X in the computational cost of the localization algorithm when compared to an algorithm that uses the sampled microphone signals (Liu et al., 2014).

To further understand the possible benefits of this event-driven sensory representation, extensive databases recorded from these sensors will be useful for research that show the comparative advantages of methods for processing spikes from these bio-inspired sensors. This work uses a database of spike recordings from a particular silicon spiking cochlea system (AEREAR2) in response to recordings from the TIDIGIT database used in many speech studies.

The AEREAR2 system (Liu et al., 2014) is one of a number of silicon cochlea designs which embed a subset of properties of the biological cochlea ranging from the spatial frequency-selective filtering of the basilar membrane, the rectification property of the inner hair cells, the local automatic gain control mechanism of the outer hair cells, and the generation of spikes by the spiral ganglion cells (Lyon and Mead, 1988; Watts et al., 1992; Lazzaro et al., 1993; van Schaik et al., 1996; Kumar et al., 1998; Abdalla and Horiuchi, 2005; Fragniere, 2005; Georgiou and Toumazou, 2005; Sarpeshkar et al., 2005; Chan et al., 2006; Katsiamis et al., 2009; Wen and Boahen, 2009; Liu and Delbruck, 2010; Liu et al., 2014). In these silicon implementations, certain cochlea models are preferred for their ease of design in VLSI technology. The first VLSI cochlea by Lyon and Mead (1988) implements the all-pole filter cascade (APFC) model of the basilar membrane even though other cochlea models like the gamma-tone filter bank can explain better the results of various auditory psychophysical experiments such as the two-tone masking experiments (Irino and Patterson, 2005).

The binaural 64-channel AEREAR2 cochlea model also uses the APFC circuit of Lyon and Mead (1988) as the base for the front-end with subsequent circuits for modeling the inner hair cell and the spiral ganglion cells. The spike outputs of this sensor system have been applied in various auditory tasks such as digit recognition (Abdollahi and Liu, 2011), speaker identification (Chakrabartty and Liu, 2010; Liu et al., 2010; Li et al., 2012), source localization (Finger and Liu, 2011; Liu et al., 2014), and sensory fusion (Chan et al., 2006, 2012; O'Connor et al., 2013). The analogous APFC model is described by the Lyon cochlea model (Lyon, 1982) implemented in Matlab within a widely used toolbox by Slaney (1998).

Besides the creation of this new database in this work, the second aim is to reconstruct the acoustic input from the spike responses in this database. Reconstructing the audio is a way of studying the fidelity of the information carried by the spikes from a particular model. Theoretical methods exist for reconstruction, for example, methods to recover the analog stimulus that stimulate biological neurons such as retinal ganglion cells. They include the use of a generalized linear integrate–and-fire neuron (Pillow et al., 2005) or non-linear methods for reconstructing the analog input to retinal ganglion cells (Warland et al., 1997).

Other studies demonstrate that the fidelity of the reconstructed analog input to an integrate-and-fire neuron model is high if one ensures that the frequency range of the analog input is restricted (Lazar and Toth, 2004; Lazar and Pnevmatikakis, 2008). This study is based only on the threshold non-linearity of the neuron and would have to be adapted to include other non-linearities such as the rectification property of the inner hair cell. These methods can be computationally very expensive in the hardware case because of the presence of multiple non-linearities in the model and the variance in the operating parameters of the hardware implementation due to the silicon fabrication process (Kinget, 2005).

This work presents a simpler method of reconstructing the acoustic input from the hardware AEREAR2 cochlea spikes by using an optimal reconstruction method (Mesgarani et al., 2009) previously proposed for reconstructing the auditory stimulus from the recorded spike responses in the ferret auditory cortex. It has also been applied successfully to other types of neural recordings, including local field potentials (Pasley et al., 2012a) and scalp-EEG (O'sullivan et al., 2014). This linear reconstruction method is computationally cheaper than non-linear methods and establishes a lower-bound on the stimulus information that is encoded in the spike outputs of the cochlea models.

This reconstruction method is applied to the spikes from both the hardware AEREAR2 system and the software Lyon cochlea model. The quality of the reconstructed acoustic waveform from both the hardware and software cochlea spikes is evaluated using the Perceptual Evaluation of Speech Quality (PESQ; Hu and Loizou, 2008) and Short Time Objective Intelligibility score (STOI; Taal et al., 2011) tests. In addition, the reconstructed audio is tested on a word recognition task and compared against the original audio of the TIDIGIT database in different SNR conditions by adding babble noise.

Section 2 describes the cochlea models and methods used in the reconstruction work, Section 3 describes the results of the perceptual tests and the digit classification task on the reconstructed audio from the software and hardware cochlea models and Section 4 discusses the results.

## 2. Materials and methods

This section covers the details of the software and hardware spiking cochlea models, the audio reconstruction algorithm from the cochlea spikes, and the subjective performance evaluation tests on the reconstructed audio and finally the use of this audio in a digit recognition.

### 2.1. Cochlea models

The cochlea architecture used in both the software and hardware cases consists of a cascaded set of filters with a log spacing of center frequencies.

#### 2.1.1. Software cochlea

We use the Lyon's cochlear model implemented using the Slaney toolbox (Slaney, 1998). This early stage model has also been used in other auditory computational models (Yildiz et al., 2013). The analog filter outputs of the cochlea stages (or channels) are used as the firing probabilities of neurons for each channel of the cochlea in a particular time bin, *n*. The matrix describing the firing probabilities of the channels vs. the time bins is called a cochleagram *C*. The default values of the parameters from the toolbox are used: the ear quality factor = 8, and the step factor (or window overlap) between two filters is 0.25. To match the hardware AEREAR2 cochlea model, we chose 64 filters which cover the frequency range from ~63 Hz to 10.7 KHz and did not include the Automatic Gain Control factor.

**Figure 1:**
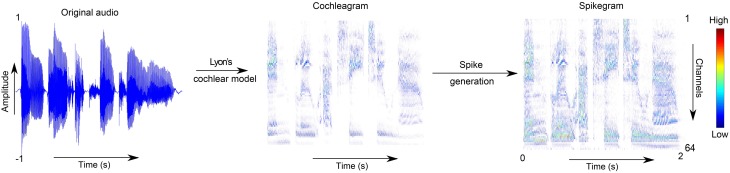
**Transformation from audio to spikegram**. This figure shows the steps of first transforming the audio into a cochleagram using the Lyon's cochlea model in Slaney's toolbox, and second, using the analog values of the cochleagram as probabilities for generating the spikegram.

The matrix of spikes (or spikegram) in response to a particular sound is generated by comparing the firing probabilities of the corresponding cochleagram to a random matrix:

(1) $$\begin{array}{l} P\left[t,m\right]=\left\{\begin{array}{cc} 1 & C\left[t,m\right]\geq M\left[t,m\right]\\ 0 & C\left[t,m\right]<M\left[t,m\right] \end{array}\right\} \end{array}$$

where *m* = 1…64 is the cochlea channel index, *t* = 1…*T* is the index of the time bin, and *M* is a matrix where each element takes a random value in the range [0,1]. The time bin of the spikegram *P* is the same as that of *C*, that is, *n* = 1∕*F*_*s*_ = 0.05ms, where *F*_*s*_ is the sampling rate of the audio signal. A smoother version of the spikegram is computed by generating a *P* matrix over several trials and then computing their averages, $\;P_{k}=\frac{1}{k}\sum_{i=1}^{k}P_{i}$. The values *k* = 1, 10, 100 are used in this work. Theoretically, averaging over infinite number of trials will produce the distribution of the cochleagram. An example of how the original audio sample is transformed into a cochleagram and then into a single trial of a spikegram is shown in Figure 1.

#### 2.1.2. Hardware cochlea

**Figure 2:**
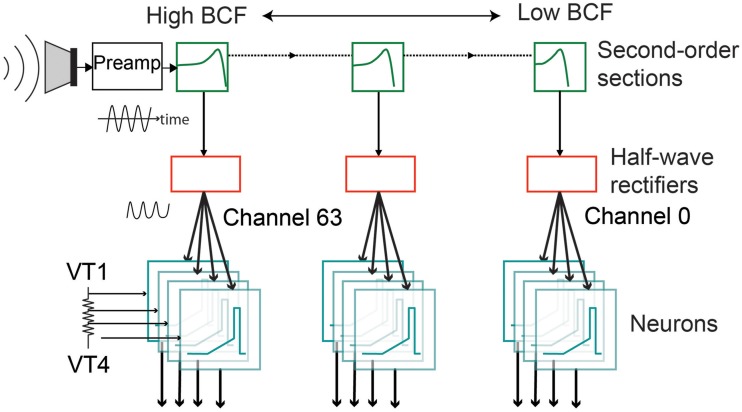
**Architecture of one ear of the spike-based binaural AEREAR2 system**. The binaural cochlea has 64 channels per ear. Each channel has a model of the basilar membrane (second-order section), a model of the inner hair cell (modeled by half-wave rectifier), and four spiral ganglion cells (modeled by integrate-and-fire neurons) driven by four separate thresholds, VT1–VT4. The incoming output of the microphone goes to a preamplifier before the audio is processed by the different channels of the cochlea, starting with the filters with the highest best characteristic frequency (BCF). Adapted from Liu et al. (2014).

**Figure 3:**
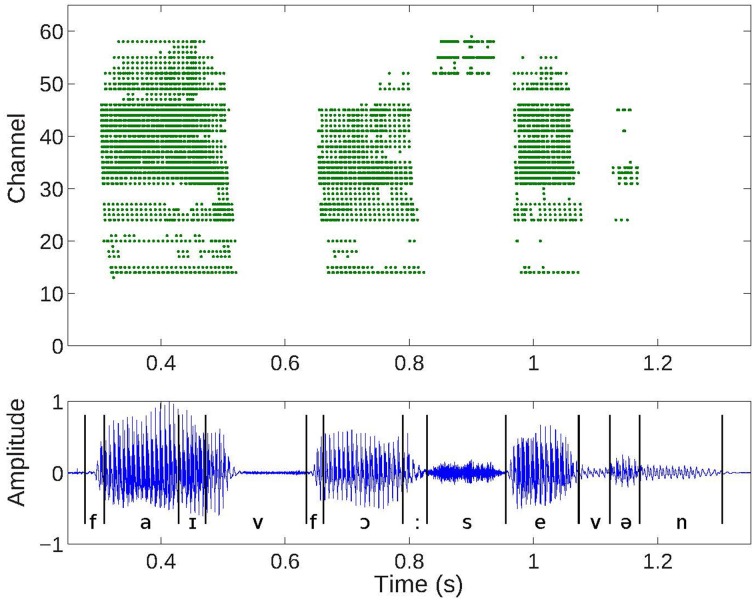
**Cochlea spike responses to a digit sequence “5, 4, 7” (top) and the corresponding audio waveform (bottom)**. Events or spiral ganglion cell outputs from the 64 channels of one ear of the binaural AEREAR2 chip. Low channels correspond to low frequencies and high channels to high frequencies.

The silicon AEREAR2 cochlea system comprises a binaural cochlea each modeled as a 64-stage cascaded filter bank receiving inputs from two microphones (right and left ear). The cascaded filter bank models, the basilar membrane and the filter outputs are then rectified by an inner hair cell, and spiral ganglion cells with details in Chan et al. (2006) and Liu et al. (2014). Each stage of the cascaded filter bank corresponds to one channel and comprises a second order section which models the basilar membrane, followed by a half-wave rectifier modeling the inner hair cell, and a spike generator modeling the spiral ganglion cell. The cochlea architecture is shown in Figure 2 and follows the architecture of the basilar membrane silicon design of the Lyon's model first described in Lyon and Mead (1988). A sample response of the spike train generated by the AEREAR2 in response to a speech sample is shown in Figure 3. In this plot, the best characteristic frequencies (BCF) of the 64 filter channels have been tuned from 50 Hz to 20 kHz. The high frequency channels do not generate spikes because of both the chosen volume of the recorded audio and the lower energy in high frequency signals. The sharpness of the filter is described by its quality factor, Q. Because this cochlear architecture depends on the cascading of the filters to build up the effective Q of each filter, the initial filters of the high frequency channels do not have enough effective Q to amplify the inputs.

### 2.2. Reconstruction algorithm

Stimulus reconstruction (Fairhall et al., 2001; Mesgarani et al., 2009) is an inverse mapping method in which the neural responses are used to approximate the acoustic representation of the sound that was heard by a subject. It is applied in brain-computer decoding applications (Pasley et al., 2012a; O'sullivan et al., 2014). This method can be used to investigate what stimulus features are encoded in the neural responses (Borst and Theunissen, 1999) by examining the neural information in the stimulus space, where it is better understood. To use this method in our study, spikes from either the software or hardware cochlea models are binned to convert them into a spike histogram (or binned spikegram). The reconstruction method then finds a linear mapping from the spikegram at each time to the time-frequency representation of the sound that elicited the spikes. Once this mapping is learned, any unseen binned spikegram can be transformed to the corresponding auditory spectrogram. Since the auditory spectrogram does not contain the phase information, a convex projection algorithm is used to recover the phase signal that is needed to reconstruct the audio from the estimated auditory spectrogram (Chi et al., 2005).

#### 2.2.1. Auditory spectrogram from audio

The auditory spectrograms are computed using the MATLAB implementation of the NSL Auditory Model toolbox from the University of Maryland (Chi and Shamma, 2005). Spectral analysis of a sound is performed in the time domain using a bank of auditory filters logarithmically placed along the frequency range of 180–7246 Hz after the waveforms are resampled at 16 kHz. Both the time constant for the leaky integration and that of the frame length are set to 10 ms. The auditory spectrogram in this study has 128 channels and a 10 ms resolution. The resulting time-frequency representation mimics the early auditory nerve responses.

#### 2.2.2. Mapping algorithm

Before the reconstruction step, the spikes from both the hardware and software cochlea models are first binned using 1 ms bins and further resampled into 10 ms time bins resulting in a binned spikegram. Then, the mapping is carried out using the “optimal stimulus prior” method originally derived to reconstruct the stimulus from the auditory cortex neural responses (Mesgarani et al., 2009). This method estimates the optimal linear mapping between the binned spikegram *R*(*t, m*) and the stimulus auditory spectrogram by minimizing the mean-squared error (MSE) between the original S(*t, f*) and reconstructed spectrograms Ŝ(*t, f*) [*t* = 1…*T* is the time bin, *m* = 1…64 cochlea channel index, and *f* = 1…128 is the frequency bin of the auditory spectrogram]. The mapping takes into account past as well as future time bins with a lag τ up to 15 time bins. Thus, we get:

(2) $$\begin{array}{l} Ŝ\left(t,\;f\right)=\overset{64}{\underset{m=1}{\sum }}\overset{15}{\underset{\tau =-15}{\sum }}g\left(t,f,m\right)R\left(t-\tau ,m\right) \end{array}$$

where *g*(*t, f, m*) is the mapping function. This equation assumes that the reconstruction of each of the frequency channel is independent of the other channels. The reconstruction of a frequency channel follows:

(3) $$\begin{array}{l} {Ŝ}_{f}\left(t\right)=\overset{64}{\underset{m=1}{\sum }}\overset{15}{\underset{\tau =-15}{\sum }}g_{f}\left(\tau ,m\right)R\left(t-\tau ,m\right) \end{array}$$

The function *g*_*f*_ is estimated by minimizing the MSE between the original and reconstructed spectrograms for that frequency channel.

(4) $$\begin{array}{l} e_{f}\;=\underset{t}{\sum }{\left[S_{f}\left(t\right)-{Ŝ}_{f}\left(t\right)\right]}^{2} \end{array}$$

This equation can be solved analytically by using a normalized reverse correlation method (Bialek et al., 1991; Stanley et al., 1999). Further details of the method can be found in Mesgarani et al. (2009).

The power spectral density of a digit in a particular spectrogram frequency bin is determined as follows:

(5) $$\begin{array}{l} \langle {Ŝ}_{f}\rangle \;=\overset{T}{\underset{t=1}{\sum }}{Ŝ}_{f}\left(t\right)∕T. \end{array}$$

The normalized power spectral density $\;{\hat{T}}_{f}$ of the reconstructed spectrogram of the digit is then computed as

(6) $$\begin{array}{l} {\hat{T}}_{f}={Ŝ}_{f}∕\underset{f}{\sum }\langle {Ŝ}_{f}\rangle \end{array}$$

#### 2.2.3. Reconstruction of audio from auditory spectrogram

The sound is resynthesized from the output of cortical and early auditory stages using a computational procedure described in detail in Chi et al. (2005). While the non-linear operations in the early stage make it impossible to have perfect reconstruction, perceptually acceptable renditions are still feasible as demonstrated in Chi et al. (2005). The reconstructed sound is obtained from the auditory spectrogram using a method based on the convex projection algorithm proposed in Yang et al. (1992) and Chi et al. (2005). This method starts with a Gaussian distributed white noise signal and iteratively shapes its frequency components prior to the non-linearity according to the mean squared distance with the target spectrogram (Yang et al., 1992).

### 2.3. Database for reconstruction

The original audio files from the TIDIGIT training sets of both male and female speakers were used in the reconstruction experiments. These files contain digit recordings from 57 female speakers and 55 male speakers. For each speaker, there are two recordings of each digit from “1” to “9” and two recordings each for the two pronunciations of 0, namely “O” and “Z.” Furthermore, there are 55 different sequences of connected single digit recordings for each speaker. These digits are presented to the hardware AEREAR2 cochlea and the software Lyon model. The recorded spikes from each cochlea model are further divided into two sets for each speaker: Half of the recorded spike data is used for learning the reconstruction filter and this filter is then applied to the remaining half of spike data to obtain the reconstructed audio dataset which is then used for the experiments described in this work. This reconstructed dataset consists of half the number of the connected digits and one copy of each single digit.

### 2.4. Performance evaluation on reconstruction

Two well-known subjective PESQ and STOI scores, which are accepted as speech quality and intelligibility metrics, respectively, are used for evaluating the quality of the reconstructed data. They are:

1. Perceptual Evaluation of Speech Quality (PESQ) scores (Hu and Loizou, 2008) computed over the target audio signal and the reconstructed audio signal. PESQ was particularly developed to model subjective tests to assess the voice quality by human beings and is a standardized measure by the International Telecommunication Union (ITU).
2. Short-Time Objective Intelligibility (STOI) scores (Taal et al., 2011) computed over the target audio signal and the estimated audio signal. STOI is based on short time (386 ms) speech intelligibility measure and has shown good correlation with the perceived speech intelligibility.

The reconstructed audio was also tested in a digit recognition task using the Hidden Markov Model (HMM) Toolkit (HTK), which includes tools for speech analysis, HMM training, testing, and result analysis (Young et al., 2009).

The HTK converts each input waveform into a feature vector consisting of 39 Mel-frequency cepstral coefficients (MFCCs). These features are fed into a HMM with five states: a beginning state, end state, and three intermediate emitting states. Each of the five states is represented by a mixture Gaussian density. For every iteration each Gaussian is split into two distributions and the unknown parameters of the hidden Markov model are re-estimated using the Baum-Welch algorithm. At the end of the fifth iteration, each emitting state contains 16 Gaussian mixture densities.

The training set used for the HTK consists of the connected digits from the reconstructed dataset as described in Section 2.3. The test set consists of the single digit samples from the same reconstructed dataset.

## 3. Results

This section shows results from using the reconstructed audio generated from both hardware (HW) and software (SW) cochlea spikes. The reconstructed audio dataset is tested in two ways: (1) through objective measures for assessing the speech quality and intelligibility and (2) through the performance on a digit recognition task.

### 3.1. Audio reconstruction

**Figure 4:**
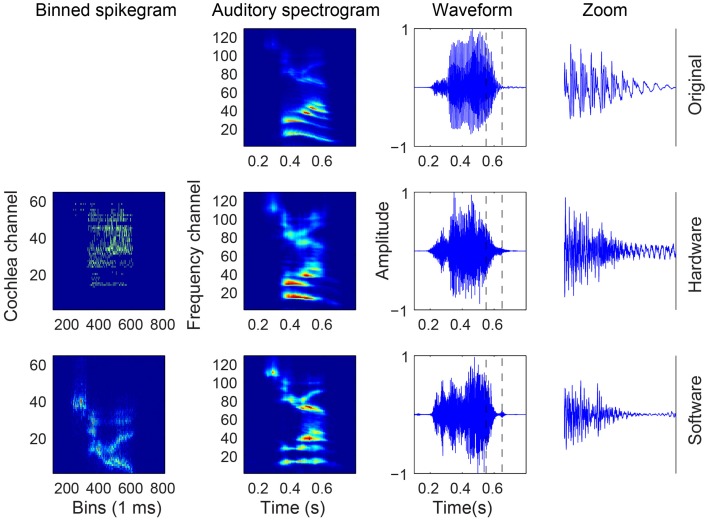
**Audio, binned spikegram, and auditory spectrogram plots for an example of digit “Z” from a female speaker (speaker FC, digit ZB)**. The responses of the software model come from a spikegram generated over 10 trials. The rightmost waveforms are zoomed-in parts of the entire waveform (third column from the left) in the region demarcated by the two vertical slashed lines. The binned spikegrams and auditory spectrograms are normalized in this figure.

The outcome of the different steps in generating the binned spikegrams of the spike responses, the mapped auditory spectrograms, and the reconstructed audio from both HW and SW spikes in response to a particular digit is presented in Figure 4. The binned spikegrams are mapped using *g*(*t, f, m*) to the corresponding auditory spectrograms which look very similar to the auditory spectrograms of the original audio. Using the NSL toolbox, the inversion of the auditory spectrogram to the audio waveform is carried out as outlined in Section 2.2. By visual inspection the reconstructed waveforms show similar temporal modulations as the original audio. The quality of the reconstructed audio is tested in speech intelligibility tests as will be described in the next section.

### 3.2. Subjective performance using PESQ and STOI metrics

**Figure 5:**
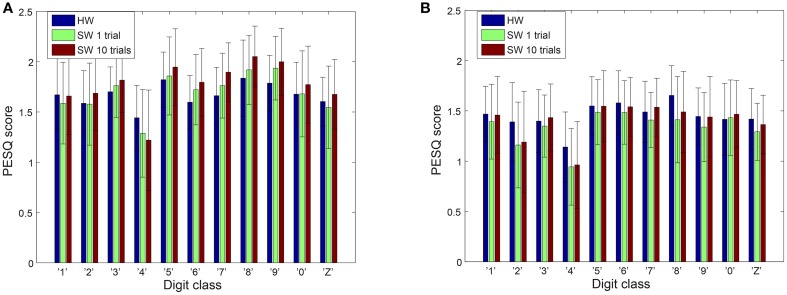
**PESQ scores on reconstructed audio**. **(A)** Male speakers **(B)** Female speakers. The error bars show the standard deviation of the PESQ scores.

The speech quality and intelligibility of the reconstructed audio from the spikes of both software and hardware cochlea models are tested using the PESQ and STOI metrics. The PESQ plot in Figure 5 shows the results from the 11 reconstructed digit classes for both male and female speakers. In both sets of speakers, digit “4” scores lower than all other digits and there is a large variation in the scores for any one digit. The possible reasons for the low score of this digit will be discussed later. Overall the different cochlea models have comparable scores.

**Figure 6:**
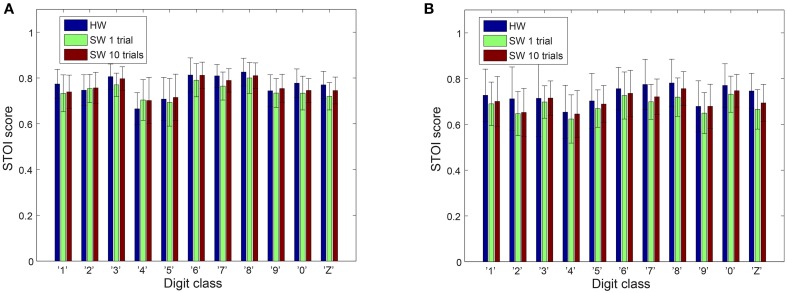
**STOI scores on reconstructed audio**. **(A)** Male speakers **(B)** Female speakers. The error bars show the standard deviation of the STOI scores.

However, better speech quality (higher PESQ scores) might not necessarily reflect better speech intelligibility. Some speech processing algorithms achieve a significant improvement in quality but this improvement is not accompanied by an increase in intelligibility (Goldsworthy and Greenberg, 2004). Therefore, it is noteworthy to also perform an intelligibility test for our results. We use the STOI metrics, which is based on the linear correlation between a time-frequency representation of the clean and reconstructed audio. STOI provides a very high correlation (>0.9) with the intelligibility scores provided by human listeners (Taal et al., 2011; Gómez et al., 2012) allowing us to make reasonable conclusions about the reconstructed audio. As can be seen in Figure 6, both the hardware and the software models have comparable scores. The *p*-values of a two-tailed *t*-test on the differences in PESQ scores as well as differences in STOI scores between hardware and software models does not demonstrate clear dominance of one model over the other.

**Figure 7:**
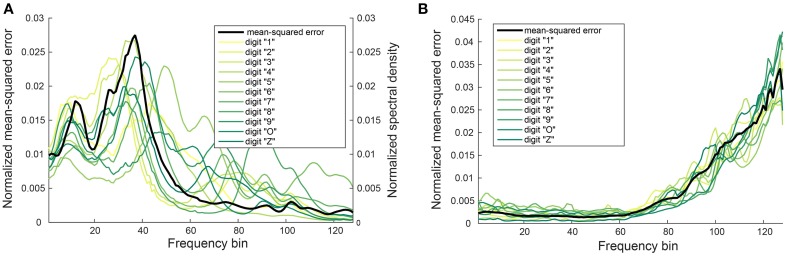
**Dependence of normalized mean-squared error on frequency bin**. **(A)** Normalized mean-squared error *e*_*f*_ in the mapping function *g*_*f*_ for each frequency bin averaged over all digits (black curve) and the normalized power spectral density of digits “1” to “Z” (light yellow to green curves). **(B)** Normalized mean-squared error *e*_*f*_ normalized by the power in each frequency bin plotted for each individual digit. The black curve shows the average over all digits. Data is extracted from the male speakers and the software cochlea model (10 trials). Ratio of neighboring frequency bins is 1.029. Minimum frequency is 180 Hz, maximum frequency is 7240 Hz. Bin 10 corresponds to a frequency around 240 Hz, bin 40 to a frequency around 571 Hz, and bin 60 to a frequency around 1017.5 Hz.

We found that certain digits (for example, digit “4”) have a lower PESQ or STOI score. This could be due to a larger error in the mapping function for certain frequency bins. The reconstructed digits whose power is primarily concentrated in these frequency bins would also have a larger error in the difference between their mapped auditory spectrogram and the corresponding spectrogram of the original audio. To determine if this is one of the reasons for the lower scoring, we plotted the normalized mean-square error *e*_*f*_ for each frequency bin across the 11 digits as shown in Figure 7. The frequency-dependent error is maximal for frequency bins between 20 and 40 but when normalized by the power in the individual bins, the maximum shifts to the higher frequency bins (Figure 7B).

**Figure 8:**
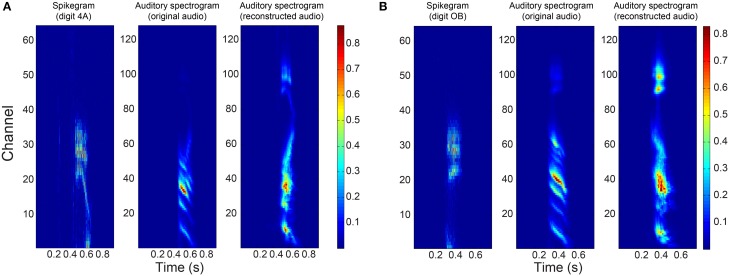
**Responses to two example digits**. Binned spikegrams (first column), auditory spectrograms of the original audio (middle column) and of the reconstructed audio (last column) for **(A)** digit “4” and **(B)** digit “O”.

How this error in the mapping function manifests itself in the auditory spectrogram can be seen in Figure 8 where the reconstructed spectrograms of two example digits show a big difference to the spectrograms of the original audio in the higher frequency bins. In the case of digit “4,” we see increased power in these frequency bins of its reconstructed spectrogram. The increased power leads to distortion in the reconstructed audio and could be a reason for its “poor” PESQ score. Even with perceptual distortions in the audio during listening, the reconstructed audio could still form a good representation for the digits in a word classification task. In Section 3.3, we describe experiments that test the performance of the reconstructed audio in a digit recognition task.

### 3.3. Classification performance on digit recognition task

There is a remarkable difference between the classification accuracies on reconstructed digits from the HW and SW models when the training set comes from the original audio versus when it comes from the reconstructed audio as shown in Table 1. When the training set is composed of the original audio samples of the TIDIGIT database, the test performance on the reconstructed audio is quite poor. However, when the reconstructed audio samples from the source cochlea models are used for the training set, the performance increased dramatically. For example in the case of the HW cochlea spikes of the female speakers, the accuracy increased from 28.2 to 88.1%. A similar increase in performance is also seen in the reconstructed audio of the SW cochlea spikes. This increase in performance is in line with results from Hirsch and Pearce (2000) that demonstrated an increase in classification accuracy for noisy digits when noisy digits instead of clean digits are used for the training set. In general, the classification accuracy using the source reconstructed audio in both the training and testing sets is higher for the HW cochlea spikes when compared to the accuracy of the spikegram created from one trial using the software cochlea model. In the three SW cases that we consider here, the classification accuracy of the hardware model is closest to that of the software model where the averaged spikegram is generated over 10 trials.

**Table 1:** **Classification accuracy of spikes from both hardware (HW) and software (SW) cochlea models using original and reconstructed audios for the training set**.

	**F/M**	**Test**				
		**Original**	**HW**	**SW 1 trial**	**SW 10 trials**	**SW 100 trials**
Train	Original	**99.8/100**	28.2/ 33.1	23.8/46.5	29.2/54.9	31.1/43.3
	HW	92.7/85.5	**88.1**/**79.3**	78.5/73.7	84.9/79.7	84.5/81.5
	SW 1 trial	90.3/82.3	71.9/61.3	**79.3**/**80.3**	89.8/86.3	87.9/84.0
	SW 10 trials	89.3/77.9	71.5/63.1	87.2/84.1	**91.2**/**89.4**	90.1/87.9
	SW 100 trials	93.0/77.7	71.8/65.0	89.3/83.8	90.9/89.9	**90.9**/**88.6**

*Results are shown for female (F) and male (M) speakers. The entries in bold show the results when the reconstructed audio in both the training and test sets come from the same cochlea model*.

To determine whether the classification accuracy is affected by the large variability in performance between certain digits seen in the PESQ and STOI results, we also look at the classification accuracy of the individual digits generated from all cochlea model variants as shown in Table 2. The results are shown for the case where the HMM is trained using the reconstructed audio from the corresponding cochlea model spikes. We see that certain digits are classified with high accuracy, for example, digits “7” and “Z” in the case of female speakers. There is also a difference in the classification accuracy depending on the cochlea model. In the case of the hardware spikes, digits like “O” and “9” have a lower classification accuracy but digit “9” is classified with higher accuracy on the reconstructed audio from the software spikes. In contrast, digit “4” is classified with higher accuracy for the hardware audio reconstruction compared to the software model spikes.

**Table 2:** **Percentage of correctly classified digits for the different cochlea spike models**.

**F/M**	**1**	**2**	**3**	**4**	**5**	**6**	**7**	**8**	**9**	**O**	**Z**
HW	93.1/90.9	86.2/47.3	93.1/90.9	84.5/85.5	94.8/89.1	94.8/63.6	98.3/100	94.8/85.5	72.4/61.8	56.9/58.2	100/100
SW 1 trial	87.7/89.1	71.9/98.2	84.2/98.2	82.5/54.5	80.7/78.2	100/96.4	98.2/100	86/94.5	84.2/60	10.5/47.3	86/96.4
SW 10 trials	93/89.1	89.5/87.3	94.7/72.7	75.4/72.7	94.7/94.5	98.2/100	100/100	98.2/100	94.7/89.1	64.9/56.4	100/98.2
SW 100 trials	87.7/90.9	87.7/76.4	96.5/69.1	77.2/69.1	96.5/92.7	96.5/100	100/100	98.2/98.2	93/83.6	66.7/63.6	100/100

*Results are shown for female (F) and male (M) speakers*.

**Figure 9:**
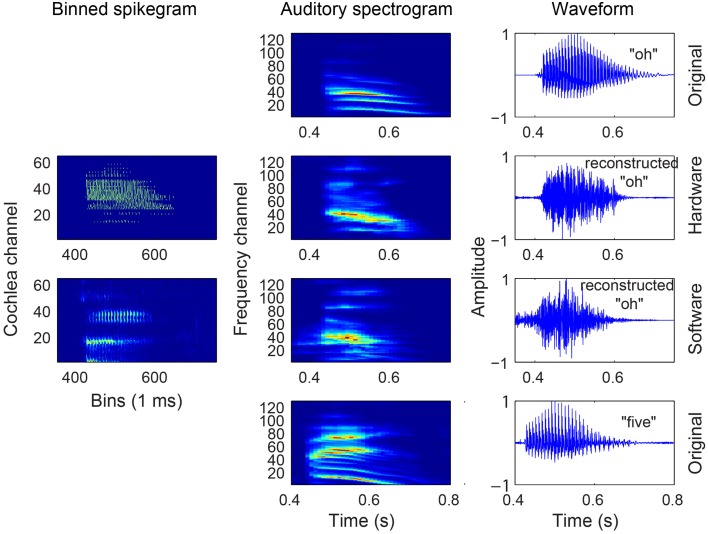
**Audio, binned spikegram, and auditory spectrogram plots for an example of a misclassified digit (male AW, digit OB)**. This digit is misclassified as digit “5” for both software and hardware spikes. The mapped auditory spectrograms for both cochlea model spikes do not resemble well the auditory spectrograms of “O” and is confused with the digit “5.” The binned spikegrams and auditory spectrograms are normalized.

For many of the digits, the classification accuracy is reasonably matched between the reconstructed audio of the HW and SW cochlea spikes. Between the two sets of speakers, we see that certain digits like “2” and “6” from the male speakers are not classified with as high accuracy as the same digits spoken by the female speakers. When listening to these incorrect classified digits, one finds that they are hardly recognizable by ear. For example, some samples sound like two digits spoken at the same time or they sound completely like another digit (see Supplementary Material). The binned spikegram, auditory spectrogram, and reconstructed audio are plotted for both cochlea models in the case of a correctly classified digit (Figure 4) and an incorrectly classified digit (Figure 9). The results in Figure 8 also explain the reason for the poorer recognition of certain digits, for example, digit “4” which has a lower accuracy and is for example, recognized frequently as digit “O.” The reconstructed spectrograms for these digits show a remarkable similarity to each other.

#### 3.3.1. Robustness to noise

**Figure 10:**
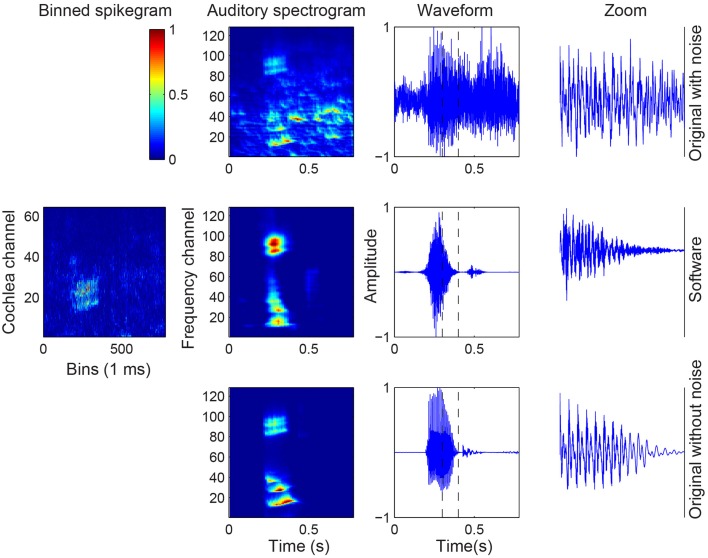
**Audio, binned spikegram, and auditory spectrogram plots for an example of a noisy digit with SNR of –10 dB**. The mapped auditory spectrograms for the cochlea model spikes resemble better the auditory spectrograms of the original clean digit (bottom row). The binned spikegrams and auditory spectrograms are normalized.

The robustness of the spike responses of the cochlea models in response to noise in the recordings is tested by creating different signal-to-noise ratio (SNR) datasets by adding babble noise to the original audio of the TIDIGIT database. The mapping function from the subsequent binned spikegrams of the noisy audio to the audio spectrograms are recomputed and the classification is tested with a network trained on both the original audio and the reconstructed audio from the SW cochlea spikes. The results for SNR of 10 dB and 5 dB show that the accuracy of the original noisy digits was still close to 100% but with decreasing SNR, the reconstructed audio maintains its accuracy much better than the original noisy audio (see Table 3). Similar to the results in Table 2, the results in Table 3 show that the performance increases significantly when the training set consists of the original noisy audio when testing the original digits and when the training set consists of the reconstructed noisy audio when testing the reconstructed audio. The results show that the original audio shows better classification results than the reconstructed audio at SNR down to 0 dB but worse performance when the SNR drops to –5 dB and even worser when the SNR is –10 dB. To understand the reason for the higher performance of the reconstructed audio from the noisy wave files, we plot the mapped auditory spectrogram from the spike responses to the noisy audio digit (Figure 10). Interestingly, the mapped auditory spectrogram contains less noise in the time bins which are not occupied by the original digit suggesting that the thresholding in the spike generation mechanism played a role in reducing noise.

**Table 3:** **Classification accuracy averaged over female (F) and male (M) speakers using either the original audio, noisy audio, or the reconstructed audio from the SW cochlea spikes (spikegrams averaged over 10 trials) at SNR values of 0, –5, and –10 dB**.

**F/M**	**Original audio SNR**			**Reconstructed audio SNR**			
**Training set**	**0 dB**	**–5 dB**	**–10 dB**	**Clean**	**0 dB**	**–5 dB**	**–10 dB**
Original audio	33.8/28.3	15.4/14.2	10.5/9.4	29.2/54.9	23.9/20.7	17.4/17.4	12.4/14.2
Noisy original/reconstructed audio	91.2/94.6	41.6/55.6	15.6/16.4	91.2/89.4	70.0/74.0	56.3/59.8	38.0/42.0

## 4. Discussion

Understanding the fidelity of the reconstructed audio from the spike trains of the AEREAR2 cochlea spikes can offer insights into the results from various auditory tasks that use the hardware spiking cochlea such as speech recognition (Verstraeten et al., 2005; Uysal et al., 2006, 2008; Chakrabartty and Liu, 2010), speaker identification (Chakrabartty and Liu, 2010; Liu et al., 2010; Li et al., 2012) localization (Finger and Liu, 2011; Liu et al., 2014), and sensory fusion (Chan et al., 2012; O'Connor et al., 2013).

Although there is prior theoretical work that demonstrates that the inversion can be done faithfully on the Lyon cochlea model, this reconstruction is only performed on the analog outputs of the cochlea (Cosi and Zovato, 1996) and not on spikes. The theoretical methods proposed for reconstructing an analog signal faithfully from the spike outputs of an integrate and fire neuron (Lazar and Toth, 2004; Wei and Harris, 2004; Wei et al., 2006; Lazar and Pnevmatikakis, 2008) could not be easily extended to the hardware VLSI cochlea spikes because of the non-linearities in the acoustic preprocessing and the silicon mismatch inherent in VLSI circuits.

This work presents instead an alternate approach for reconstructing the acoustic input from the cochlea spikes based on the optimal prior reconstruction method proposed by Mesgarani et al. (2009). This method learns a linear mapping from the binned spikegram of the cochlea spikes to the auditory spectrogram of the acoustic input.

The results of this mapping showed that even though the PESQ and STOI scores of the reconstructed audio were not very high, a fairly good reconstruction is still possible as evident when listening to the reconstructed digits. We also investigated the performance from the use of the reconstructed audio in a task-specific problem, such as digit recognition which does not require a perceptual understanding of the acoustic input. The results were good for many of the digits (98 to 100% accuracy). Our results also show that the accuracy goes up significantly when the reconstructed audio was used for the training set rather than the original audio most likely because the statistics of the training and testing sets were more similar. The poor recognition of the digits could also be due to the possibility that the performance of HTK generally goes down significantly with noise in the data or if both training and testing data comes from extremely different data sets (e.g., different accents; Chengalvarayan, 2001). Thus, the noise and the distortions in the reconstructed sound can affect the recognition output of the HTK toolbox even though the digits are still discernable by ear.

One interesting outcome of our work is the similar performance obtained with both the reconstructed audio from the hardware AEREAR2 cochlea and that from the software model (with spikegrams averaged over 10 trials) in almost all the presented results, therefore suggesting that the use of the hardware cochlea in various auditory tasks mentioned at the beginning of this section can be validated by an equivalent software model. The results in this paper also suggest that there could be improvements on the method for transformation of the acoustic input to spikes. We did not, for example, use the gain control parameter in the Lyon-Slaney cochlea model therefore spikes were not generated in places of the audio with low amplitudes. Other cochlea models such as the gamma-tone filterbank design (Patterson, 1976) and the CAR-FAC model (Lyon, 2011), with an added spike-generating block could lead to better reconstruction results and give guidance for hardware implementation of these models (Katsiamis et al., 2009; Thakur et al., 2014). Adding companding strategies to the cochlea model could also help to preserve information in the acoustic input under low SNR conditions as illustrated by results from vowel-in-noise experiments (Turicchia and Sarpeshkar, 2005).

Another area of improvement is the use of alternate methods to the linear reconstruction method proposed in this work. For example, better results can be obtained from the use of a non-linear model such as the one described in Warland et al. (1997). The results can be validated in the future on the spike TIDIGIT database created within this benchmarking study.

Another interesting result of this study is the higher classification performance of the reconstructed audio over the original audio in the digit recognition task for SNR-values below –5 dB. These results are in line with other studies that show for example, the better classification performance of phonemes using a cochlea model over MFCCs (Jeon and Juang, 2007) and better noise rejection (Mesgarani et al., 2006). Reservoir networks with a cochlea pre-processing stage also show better classification results on speech recordings under low SNR conditions as previously demonstrated by Verstraeten et al. (2005) and Uysal et al. (2006, 2008).

This work serves as a benchmark for future hardware cochlea designs and for understanding the output of other spike-based software cochlea models. The AEREAR2 spike recording database will be made available for the research community.

## 5. Author contributions

AZ performed the reconstruction mapping on the cochlea spike recordings, the digit recognition task, and data analysis. SB performed the software cochlea model recordings, generation of the spike responses, and data analysis. NM contributed to the conception of the work and software for the optimal reconstruction filter. SL performed the hardware cochlea recordings and contributed to the conception and design of the experiments, data analysis, and presentation of the work.

### Conflict of interest statement

The authors declare that the research was conducted in the absence of any commercial or financial relationships that could be construed as a potential conflict of interest.

## Supplementary material

The first and second audio files are reconstructed from the hardware model spikes, the third and fourth audio files are reconstructed from the software model (10 iterations) spikes.
